# The relationship between hepatic resistin overexpression and inflammation in patients with nonalcoholic steatohepatitis

**DOI:** 10.1186/1471-230X-14-39

**Published:** 2014-02-23

**Authors:** Chuan Shen, Cai-Yan Zhao, Wei Wang, Ya-Dong Wang, Hui Sun, Wei Cao, Wei-Yan Yu, Li Zhang, Ru Ji, Meng Li, Jian Gao

**Affiliations:** 1 Department of Infectious Disease, The Third Affiliated Hospital of Hebei Medical University, 139 Ziqiang Road, Shijiazhuang 050051, China; 2 Department of Endocrinology and Metabolism, The Third Affiliated Hospital of Hebei Medical University, 139 Ziqiang Road, Shijiazhuang 050051, China; 3 Department of Hepatobiliary Surgery, The Third Affiliated Hospital of Hebei Medical University, 139 Ziqiang Road, Shijiazhuang 050051, China

**Keywords:** Resistin, Nonalcoholic steatohepatitis, Nonalcoholic fatty liver disease, Inflammation, Adipokine

## Abstract

**Background:**

The relationship between resistin and non-alcoholic steatohepatitis (NASH) is not clear, some studies claimed that serum resistin levels were associated with neither the presence of NASH nor its severity, others declared that serum resistin was related with inflammation and fibrosis in NASH. Our animal study verified that the distribution of resistin in the liver is correlated with inflammation in NASH. However, there is no pertinent study in humans.

**Methods:**

Thirty patients with NASH, 28 simple steatosis, and 43 controls were recruited. Blood was collected for resistin, liver chemistries, fasting insulin and some metabolic parameters. Liver histology was scored according to NAFLD activity scoring system. Hepatic resistin expression was examined by real-time polymerase chain reaction, immunohistochemistry. Resistin protein expression was confirmed by western blotting in 13 patients with concomitant NAFLD and gallstone.

**Results:**

**S**erum resistin was significantly elevated in both NASH and simple steatotic subjects compared with controls (all *P* < 0.05). Hepatic resistin was significantly increased in NASH patients in both mRNA and protein levels than those in simple steatosis and control subjects (all *P* < 0.05). Both serum and hepatic resistin had a correlation with obesity, but not with insulin resistance. The distribution of resistin positive cells was predominantly in perisinusoidal cells (such as Kupffer cells and hepatic stellate cells) in human NASH. Multivariate analysis revealed that waist-hip ratio, higher serum triglyceride, and hyperresistinemia were independent factors related to higher grade of steatosis; whereas hepatic resistin and serum cytokeratin predict NASH and severity of liver fibrosis.

**Conclusions:**

Hepatic resistin overexpression in NASH patients is associated with the severity of liver inflammation and fibrosis. Liver-derived resistin may be involved in the pathogenesis of human NASH.

## Background

Nonalcoholic fatty liver disease (NAFLD) is a clinicopathological entity with a wide spectrum ranging from simple steatosis, to steatohepatitis (NASH), to cirrhosis [1,2]. Being different to the past views, fatty liver is now regarded as an abnormal condition due to simple steatosis patients also exhibiting elevated liver enzymes in serum. However, it is widely accepted that NASH is the more aggressive form of NAFLD, which is characterized by the hepatocellular injury, hepatic infiltration of inflammatory cells. Strong evidence suggests that NASH ultimately lead to scarring of the liver (fibrosis) and then irreversible, advanced scarring (cirrhosis) [3]. The mechanism of NASH is still not clear, proinflammatory cytokines, such as tumor necrosis factor α (TNF-α) and interferon γ are involved. Recent studies found that resistin is another important cytokine to be implicated in the pathogenesis of NASH.

Resistin, also known as found in inflammatory zone-3 (FIZZ-3), is a cysteine-rich polypeptide primarily produced by white adipose tissue in rodents and monocytes or macrophages in humans [4]. Several rodent models have shown that the major target organ of resistin is liver [5,6]. However, the physiological role of resistin in humans remains controversial. Some studies claimed that serum resistin levels were associated neither with presence of NASH nor with its severity [7,8], others declared that serum resistin was related with inflammation, fibrosis and its severity in NASH [9,10]. Although resistin is recognized as a proinflammatory cytokine, the role of its local expression in the liver is not clear. Our animal study showed that resistin has direct effects on NASH: (1) resistin was increased in inflammatory liver in rats; and (2) recombinant resistin directly stimulate activation of nuclear factor kappa B and proinflammatory cytokines secretion in murine hepatocytes [11]. There is no relevant data available in human being. To verify the connection between resistin and NASH in human may eventually lead to NASH intervention.

The present study was to evaluate the circulating and hepatic resistin expression in patients with biopsy-proven NASH and those with simple steatosis, and to assess the associations of resistin expression with metabolic profiles and inflammation in human liver.

## Methods

### Study design

We prospectively studied 58 biopsy-proven NAFLD patients (30 with NASH and 28 with simple steatosis) at the Third Affiliated Hospital of Hebei Medical University between July 2008 and October 2010. NAFLD was diagnosed by ultrasound, alanine aminotransferase (ALT) and biopsy, the diagnostic criteria was the guidelines for the diagnosis and treatment of NAFLD suggested by the Chinese National Consensus Workshop on Nonalcoholic Fatty Liver Disease [12]. Exclusion criteria were as follows: a history of excessive alcohol consumption (>140 g/wk for male and >70 g/wk for female), viral hepatitis (hepatitis B and C), alcoholic liver disease, autoimmune hepatitis, primary biliary cirrhosis, primary sclerosing cholangitis, Wilson’s disease, hemochromatosis, α-1 antitrypsin deficiency, biliary obstruction, secondary causes of steatosis, and drug-induced liver disease. Forty-three healthy blood donor volunteers matched for age and sex were recruited as controls: normal liver function, no signs of fatty liver on ultrasound and negative serology for viral hepatitis. Seven liver samples from patients with cavernous hemangioma were also involved in this study. None of the participants had history of primary hypertension, cardiovascular disease, malignancy, hypo- or hyperthyroidism and the use of thiazolidinediones. Previously diagnosed type 2 diabetes patients were also excluded from this cohort.

Written informed consent was obtained from all participants prior to enrollment. And the study protocol was reviewed and approved by the Ethics Committee of the Third Affiliated Hospital of Hebei Medical University.

### Clinical and biochemical evaluations

Anthropometric parameters including weight, height, waist circumference, and hip circumference were measured. Body mass index (BMI) was calculated as the body weight (kg) divided by the square of the height (m). Waist-hip ratio (WHR) was calculated as the waist circumference (cm) divided by the hip circumference (cm). A venous blood sample for determination of ALT, aspartate aminotransferase (AST), γ-glutamyl transferase (GGT), glucose, triglyceride (TG), total cholesterol (TC), high-density lipoprotein cholesterol (HDL-C) and low-density lipoprotein cholesterol (LDL-C) was collected in each participant following a 12-hour overnight fasting. Serum insulin was determined by a radioimmunoassay technique. The homeostasis model assessment of insulin resistance (HOMA-IR) score was calculated as the product of fasting glucose (mmol/L) and fasting insulin (μIU/mL) divided by 22.5.

### Liver tissue collection

Percutaneous liver biopsies were performed in 58 NAFLD patients. A sample was considered valid for the study if it was at least 1.5 cm in length. Since insufficient amount of liver tissues by percutaneous liver biopsies, a few representative liver tissues for western blotting were also obtained in 13 patients with NAFLD (5 with NASH and 8 with simple steatosis) during laparoscopic cholecystectomy due to gallstone. For further comparison, seven samples of relatively normal liver tissue were collected from patients with cavernous hemangioma of the liver during surgical hepatectomy. One third of each specimen was immediately stored in RNA fixer (GENEray Biotechnology, Shanghai, China) and then stored at -80°C until RNA or protein extraction. The remains were fixed in 10% formalin for histology and immunohistochemistry.

### Hepatopathological analysis

The biopsies containing at least six portal tracts were considered appropriate for evaluation. The specimens were stained with hematoxylin-eosin and Masson trichrome. Histology was reviewed by a single hepatopathologist who was blind to the clinical data. The histological scoring of NAFLD followed the NAFLD Activity Score (NAS) proposed by The Pathological Committee of the NASH Clinical Research Network [13]. The score was composed of steatosis (0 = <5%, 1 = 5% - 33%, 2 = 34% - 66%, 3 = >66%), lobular inflammation (0 = no foci, 1 = <2 foci per 200 × field, 2 = 2–4 foci per 200 × field, 3 = >4 foci per 200 × field), and ballooning (0 = none, 1 = rare or few, 2 = many or prominent). Fibrosis staging was recorded as following criteria: 0 = none, 1 = perisinusoidal or periportal fibrosis, 2 = perisinusoidal and portal/periportal fibrosis, 3 = bridging fibrosis, and 4 = cirrhosis. The score of NAS ≥ 5, 2 < NAS <5, NAS ≤ 2 were defined as NASH, “borderline”, and simple steatosis, respectively. The “borderline” patients with NAS 3 or 4 were also identified as NASH under further investigation by original Brunt criteria [14] (i.e. steatosis together with ballooning and/or Mallory-Denk bodies or fibrosis ≥ 2), while the remainder were regarded as simple steatosis.

### Real-time polymerase chain reaction (PCR)

Total RNA was extracted from the RNA fixer-stored specimen using Trizol reagent (Invitrogen, Carlsbad, CA) according to the protocol. The extracted RNA was reverse transcribed to first strand cDNA using the PrimeScript RT reagent Kit (Fermentas, Burlington, ON, Canada). Quantification of the gene expressions were carried out in a thermal cycler (ABI 7500; Applied Biosystems, Foster City, CA) using a SYBR-green RealMaster Mix kit (TianGen Biotech, Beijing, China). The PCR parameters were an initial denaturation at 94°C for 5 min; followed by 30 cycles (94°C for 45 s, 56°C for 45 s, and 72°C for 1 min). Glyceraldehyde-3-phosphate dehydrogenase (GAPDH) was used as an internal control. The sequences of oligonucleotide primers for PCR included: resistin: forward: 5′-CCA TGG AAG AAG CCA TCA AT-3′and reverse: 5′-CTG GCA GTG ACA TGT GGT CT-3′ (product size: 209 bp); and GAPDH: forward: 5′-ACC ACA GTC CAT GCC ATC ACT-3′ and reverse: 5′-TCC ACC ACC CTG TTG CTG TA-3′ (product size: 452 bp). For each sample, PCR was performed twice in triplicates, and all data were analyzed by the thermal cycler’s software to calculate the △Ct value (△Ct = Ct value of the target gene minus Ct value of the internal control gene). The level of resistin mRNA in healthy controls was assigned as a reference value of 1. Relative expression of resistin to the internal control was calculated using a 2^-△△Ct^ method [15].

### Western blotting

Liver tissues were lysed in RIPA lysis buffer with protease inhibitors according to manufacturer’s instructions (BestBio, China). The concentration of protein was measured by the Bradford method. Equal amounts of proteins (100 μg) were loaded onto 5% condensed gel and 12% SDS-PAGE gel for electrophoresis, followed by western blotting onto polyvinylidene difluoride membranes by semi-dry transfer method (Hoefer TE70X Semi-dry Bloters, USA). The membranes were blocked with 1×TBST containing 5% nonfat milk, and then incubated with both anti-resistin monoclonal antibody (1:2000; Epitomics) and anti-β-actin monoclonal antibody (1:800; Santa Cruz Biotechnology) overnight at 4°C. After washing three times, the blots were detected with Odyssey infrared imaging system (LI-COR, USA). The levels of protein were calculated as the ratio of the intensity of resistin to that of β-actin.

### Immunohistochemistry

Five μm paraffin-embedded liver sections were digested with trypsinase for 30 min at 37°C, followed by the antigen retrieval via pressure cooking for 2 min in 0.01 mol/L citrate buffer (PH 6.0). After blocking the activity of endogenous peroxidase with 3% methanol-H_2_O_2_, all sections were incubated with monoclonal antibody against human resistin (1:100 dilution; R&D Systems, USA) overnight at 4°C. After washing with 0.01 mol/L phosphate-buffered saline (PBS) (PH 7.2), Two-Step IHC Detection Reagent (Zhongshan Golden Bridge Biotech, Beijing, China) containing secondary rabbit anti-mouse antibody, and 3, 3′-Diaminobenzidine tetrahydrochloride Substrate Kit (Zhongshan Golden Bridge Biotech, Beijing, China) were applied according to the manufacturer’s protocols. The slides incubated with PBS instead of primary antibody were used as negative controls. All sections were counterstained with hematoxylin following immunochemical staining. Semi-quantitative (SQ) analysis of the average density of resistin expression was performed using a CMIAS-II morphometric analysor (Beijing University of Aeronautics and Aerospace, China). The average density was calculated by positive staining areas/total areas × 100% in a 400× high-power field (hpf) [16].

### Serum resistin and cytokeratin-18 (CK-18) levels by enzyme-linked immunosorbent assays (ELISA)

Serum levels of resistin and CK-18 fragment were measured by ELISA using commercially available kits (resistin: Rapidbio, West Hills, CA, USA; CK-18: PEVIVA, Alexis, Grunwald, Germany) according to the manufacturer’s instructions. All serum samples were analyzed in duplicates.

### Statistical analyses

All analyses were performed by SPSS version 13.0 (Chicago, IL, USA). All statistical tests were two-tailed, and *P* < 0.05 was considered to be statistically significant.

Continuous variables were expressed as means ± standard deviation (SD). Differences between groups were analyzed by One-Way ANOVA, followed by Student-Newman-Keuls test for multiple comparisons. Logarithmic transformation of data was performed when appropriate. Pearson Chi-square test or Fisher’s exact test were used to analyze categorical variables. Bivariate correlation analysis was assessed by Pearson correlation test or Spearman correlation test.

Logistic regression analysis with forward stepwise variables selection was used to demonstrate the independent predictors for the histological severity of steatosis, necroinflammation, and fibrosis. The covariates included age, gender, BMI, WHR, ALT, TG, HOMA-IR score, serum resistin, serum CK-18 fragment and positive area of hepatic resistin expression.

## Results

### Characteristics of patients and controls

The anthropometric and biochemical data are presented in Table 1. The BMI was significantly higher in the NASH patients than that in the simple steatosis subjects (*P* < 0.05), and the BMI in almost all of the NASH patients (28/30) was greater than 25; whereas no significant differences in WHR (*P* = 0.88) were observed between NASH and simple steatosis. In this cohort, ALT, AST, GGT, glucose, insulin, HOMA-IR, TG, and LDL-C were significantly increased in both NASH and simple steatosis. Both ALT and serum CK-18 fragment level were significantly higher in NASH patients than that in patients with simple steatosis (*P* < 0.05). In contrast, HLD-C was significantly decreased in both NASH and simple steatosis compared with control group (*P* < 0.05). There was no significant differences in serum TC among the three groups (*P* = 0.12).

**Table 1:** Anthropometric and biochemical characteristics of all studied subjects

	**NASH**	**Simple steatosis**	**Controls**	***P *** **value**
	**(n = 30)**	**(n = 28)**	**(n = 43)**	
Age (year)	42 ± 9	44 ± 12	45 ± 14	0.64
Sex (male/female)	19/11	19/9	29/14	0.92
Body weight (kg)	80.5 ± 10.2^a,b^	74.8 ± 10.1	62.1 ± 7.9	<0.001
BMI (kg/m^2^)	28.2 ± 1.9^a,b^	26.6 ± 2.6	22.0 ± 1.8	<0.001
Waist circumference (cm)	101 ± 10^b^	99 ± 10	84 ± 7	<0.001
WHR	0.95 ± 0.04^b^	0.94 ± 0.05	0.87 ± 0.05	<0.001
ALT (U/L)	82 ± 14^a,b^	68 ± 13	21 ± 9	<0.001
AST (U/L)	38 ± 9^b^	35 ± 8	18 ± 6	<0.001
GGT (U/L)	86 ± 14^b^	80 ± 15	28 ± 10	<0.001
Fasting glucose (mmol/L)	6.1 ± 0.6^b^	5.9 ± 0.5	4.9 ± 0.4	<0.001
Fasting insulin (μIU/mL)	10.9 ± 2.2^b^	10.2 ± 2.5	7.4 ± 2.3	<0.001
HOMA-IR score	2.9 ± 0.6^b^	2.6 ± 0.7	1.6 ± 0.5	<0.001
Triglyceride (mmol/L)	2.22 ± 0.62^b^	2.04 ± 0.51	1.13 ± 0.44	<0.001
Total cholesterol (mmol/L)	5.32 ± 0.75	5.19 ± 0.72	4.99 ± 0.57	0.12
HDL-C (mmol/L)	1.21 ± 0.23^b^	1.12 ± 0.26	1.33 ± 0.18	0.01
LDL-C (mmol/L)	3.26 ± 0.52^b^	3.23 ± 0.69	2.55 ± 0.47	<0.001
CK-18 (U/L)	391.89 ± 123.68^a,b^	277.87 ± 109.90	163.62 ± 73.52	<0.001

Results were expressed as mean ± SD.

^a^*P* < 0.05 vs. simple steatosis group; ^b^*P* < 0.05 vs. Controls.

### Liver histology

The histological findings were summarized in Table 2. Of all NAFLD patients, thirty (52%) were histologically diagnosed as NASH. Among NASH patients, eighteen (60%) had moderate-to-severe (grade 2 or 3) steatosis; 14 (47%) had mild (grade 1) lobular inflammatory, 16 (53%) moderate-to-severe (grade 2 or 3); 17 (57%) had mild ballooning, 13 (43%) had many balloon cells; 18 (60%) had none or mild fibrosis (stage 0 or 1), 12 (40%) in stage 2 or 3. Steatosis was the most prominent histological change in simple steatosis and it was milder compared with patients with NASH.

**Table 2:** Histological studies of patients with NASH and simple steatosis

	**NASH**	**Simple steatosis**
	**(n = 30)**	**(n = 28)**
Steatosis grade		
0	0 (0%)	0 (0%)
1	12 (40%)	16 (57%)
2	14 (47%)	9 (32%)
3	4 (13%)	3 (11%)
Lobular inflammation grade		
0	0 (0%)	28 (100%)
1	14 (47%)	0 (0%)
2	13 (43%)	0 (0%)
3	3 (10%)	0 (0%)
Hepatocyte ballooning grade		
0	0 (0%)	28 (100%)
1	17 (57%)	0 (0%)
2	13 (43%)	0 (0%)
Fibrosis stage		
0	6 (20%)	20 (71%)
1	12 (40%)	8 (29%)
2	9 (30%)	0 (0%)
3	3 (10%)	0 (0%)
4	0 (0%)	0 (0%)

### Increased circulating resistin concentration in NAFLD patients

**Figure 1:**
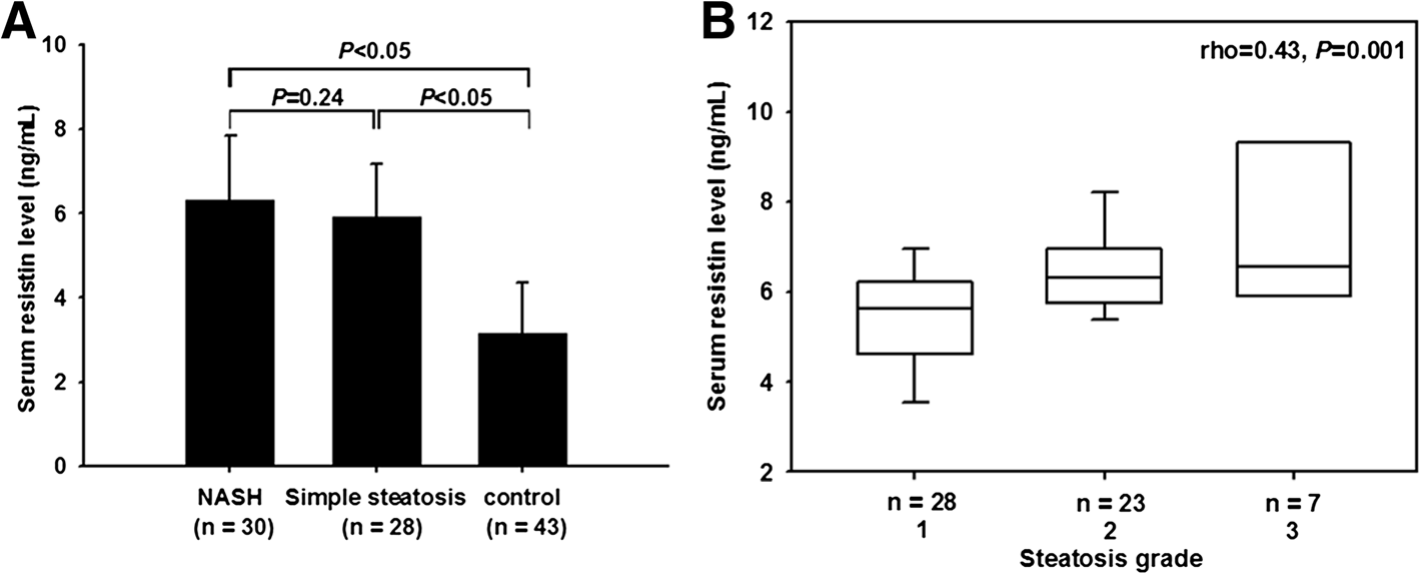
Serum resistin level was the highest in patients with NASH (A), and correlated with histological steatosis grade in the whole NAFLD cohort (B).

Serum resistin concentration in patients with NAFLD was significantly higher than that in controls (6.30 ± 1.54 vs. 3.14 ± 1.22 ng/mL, *P* < 0.05), while there was no significant difference in the subtypes of NAFLD patients (Figure 1A). Gender is not a factor to affect serum resistin in each group. Serum resistin level in NAFLD patients had a positive correlation with WHR (r = 0.41, *P* = 0.001), ALT (r = 0.31, *P* = 0.02), GGT (r = 0.35, *P* = 0.007), TG (r = 0.34, *P* = 0.01), CK-18 fragment level (r = 0.64, *P* = 0.02) and histological grade of steatosis (rho = 0.43, *P* = 0.001) (Figure 1B). There was no correlation between serum resistin and HOMA-IR score (*P* = 0.14).

### Up-regulation of resistin in liver of NASH patients

**Figure 2:**
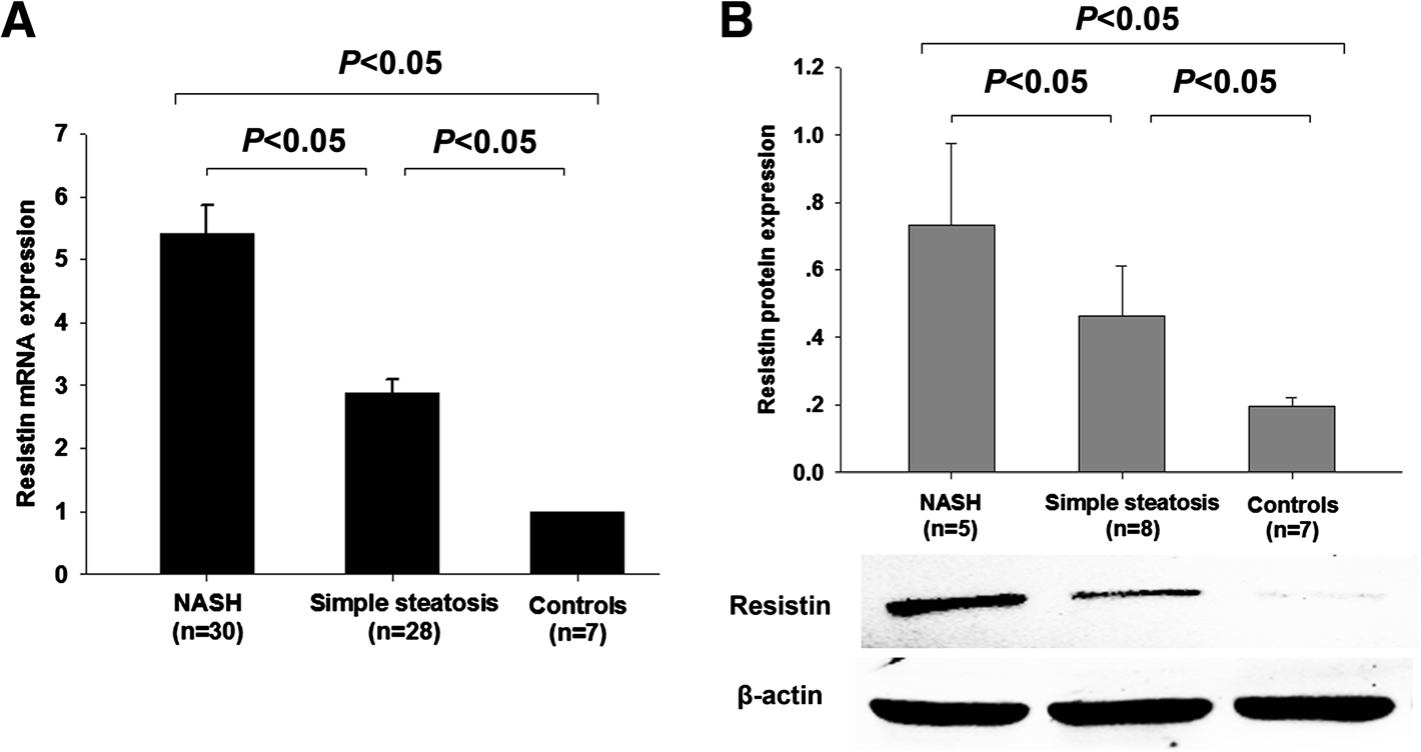
Patients with NASH exhibit increased hepatic resistin expression in mRNA (A) and ptotein levels (B) than patients with simple steatosis, and controls.

Hepatic resistin mRNA expression was significantly up-regulated in NASH patients compared with simple steatosis group (*P* < 0.05) and controls (*P* < 0.05) (Figure 2A). In parallel, NASH patients exhibited increased resistin protein expression detected by SQ-analysis than those in simple steatosis (3.12% ± 0.11% vs. 0.61% ± 0.09% per hpf, *P* < 0.05) and controls (vs. 0.30% ± 0.09% per hpf, *P* < 0.05). These SQ-analysis results of resistin protein expression were in agreement with the western blotting results from a small proportion of patients with co-existence of both NASH and gallstone, patients with concomitant simple steatosis and gallstone, and controls (Figure 2B). Nevertheless, resistin was scarcely expressed in livers of healthy controls.

### Localization of resistin in liver tissue

**Figure 3:**
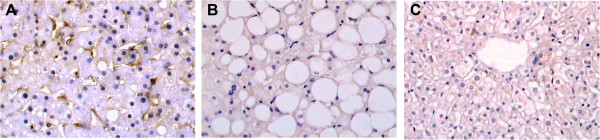
**Immunohistochemistry for resistin in human liver tissue.** Representative expression and distribution of resistin in liver tissues of patients with NASH **(A)**, patients with simple steatosis **(B)**, and controls **(C)**, respectively. (Original magnification: 400× for A and B; 200× for C).

Immunohistochemistry showed that resistin positive cells were predominantly distributed in hepatic perisinusoidal area (Figure 3), occasionally in portal tracts.

### Predictors for the histological severity of steatosis, necroinflammation, and fibrosis

**Figure 4:**
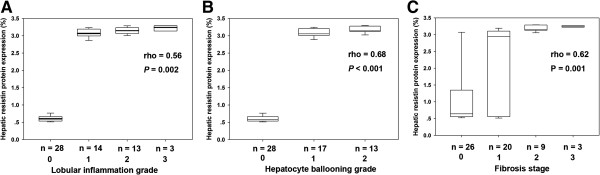
Spearman correlation analysis demonstrates that hepatic resistin protein expression correlates with lobular inflammation (A), hepatocyte ballooning (B), and fibrosis stage (C) in patients with NAFLD.

In this entire NAFLD cohort, hepatic resistin protein expression by SQ analysis was positively correlated with BMI (r = 0.38, *P* = 0.003) and ALT (r = 0.48, *P* < 0.001). Furthermore, it had a positive correlation with histological lobular inflammation grade (rho = 0.56, *P* = 0.002) (Figure 4A), hepatocyte ballooning (rho = 0.68, *P* < 0.001) (Figure 4B), and fibrosis stage (rho = 0.62, *P* = 0.001) (Figure 4C). However, there was no correlation with HOMA-IR (r = 0.22, *P* = 0.09). On logistic regression analysis, WHR (odds ratio [OR] 3.5; 95% confidence interval [CI] 1.4-9.2; *P* = 0.009), TG (OR 2.4; 95% CI 1.0-5.7; *P* = 0.045), and hyperresistinemia (OR 2.1; 95% CI 1.0-4.5; *P* = 0.045) can be used to predict moderate-to-severe hepatic steatosis. Furthermore, both serum CK-18 fragment level and amount of resistin positive cells in the liver were independent factors either to predict NASH presence (CK-18: OR 6.4; 95% CI 1.1-7.5; *P* = 0.002 and resistin: OR 4.3; 95% CI 1.9-14.3; *P* = 0.004) or moderate-to-severe liver fibrosis (CK-18: OR 5.3; 95% CI 1.6-9.2; *P* = 0.003 and resistin: OR 3.6; 95% CI 1.1-12.5; *P* = 0.012).

## Discussion

In the present study, we found that (1) resistin was significantly increased in the liver of NASH patients compared with that of subjects with simple steatosis and controls; (2) resistin distribution and its density is closely correlated with the inflammation and inflammatory severity in the liver; (3) both serum and hepatic resistin expression had correlation with obesity, but not with insulin resistance; (4) immunoreactivity for resistin was confined to hepatic perisinusoidal cells; and (5) WHR, hypertriglyceridemia, and hyperresistinemia were the independent predictors for the higher degree of steatosis, whereas increased serum CK-18 and hepatic resistin levels predicted NASH and more severe histological fibrosis.

The link between resistin and NAFLD remains under debate in humans. It is accepted that obesity, particularly central obesity is a risk factor strongly associated with NAFLD [17-19]. About half of the literatures support the notion that serum resistin increases are accountable for the obesity and insulin resistance [20-23], while the other half showed the opposite: resistin is downregulated in obese subjects and resistin had no correlation with insulin resistance [8,10,24]. Resistin is primarily secreted by monocytes or macrophages in humans [25-27]. On one hand, obesity-associated inflammation stimulates the adipocytes themselves to produce inflammatory mediators, on the other hand, these inflammation mediators aggravate inflammation and thus, increases resistin secretion and vice versa. This vicious circle causes and maintains macrophages infiltration into adipose tissue [28,29]. Our data showed that, in NAFLD patients, serum resistin correlated with WHR, and hepatic resistin protein expression correlated with BMI, whether this correlation is coincidence or casual-effect needs further investigation.

Accumulating evidence indicates that resistin has proinflammatory properties. Resistin enhances interleukin 6 and TNF-α production, both are increased in NAFLD patients [7,30-32]. Our results showed that resistin was increased in liver of NASH patients, and there was a positive correlation between resistin and inflammatory severity in the liver of NASH patients. The distribution and density of resistin positive cells was associated with the existence and severity of necroinflammation, ballooning, and fibrosis, these results are consistent with other studies [10,33]. Moreover, recent studies have demonstrated that several useful biomarkers, such as CK-18 and fetuin-A, may reflect apoptosis, necrosis of hepatocytes, as well as disease severity in NAFLD [34,35]. In this NAFLD cohort, there is a positive correlation between serum resistin and CK-18, which is largely increased in NASH patients. Thus, our and other studies might implicate that resistin had direct effect on liver inflammation and fibrosis. Since the insufficient amount of liver tissue obtained by percutaneous liver biopsy, quantitative analysis of hepatic resistin protein content has only been performed in a small number of NAFLD patients together with gall stones who are willing to provide liver tissues during laparoscopic cholecystectomy. Thus, quantitative liver resisitin protein content needs to be examined in a further larger cohort.

Another interesting finding is that serum resistin had positive correlation with hepatic fat content in NAFLD patients. Furthermore, WHR, increased serum TG, and hyperresistinemia predicted moderate-to-severe steatosis. The excess resistin found in the serum of NAFLD patients among other molecules that might metabolically be important could contribute to the speed-up of metabolic deterioration observed in NAFLD [4]. The serum resistin in NASH patients is similar with that in the subjects with simple steatosis, while resistin content in NASH liver is significantly higher than that in the liver of simple steatosis. This inconsistency might indicate that serum resistin is parallel with fat accumulation in the body, liver is one of the organs where the fat is stored. Although the serum resistin level is almost the same in NAFLD, the ALT and CK-18 are significantly increased in NASH patients compared with simple steatosis which means that it is the resistin in the liver that may initiate the progression from simple steatosis to NASH and it is the resistin in the liver that correlated with inflammation and fibrosis and, damage the liver tissue to release biomarkers of liver injury.

We further identified the cell types in liver responsible for the production of resistin by immunohistochemistry. Our findings showed that resistin protein expression was mainly localized in perisinusoidal cells. It is proposed that the liver harbors two types of cells that are particularly attractive candidates for resistin production—Kupffer cells (i.e. resident liver macrophages) and hepatic stem cells (HSC) (i.e. adipocyte-like cells), which exert their physiological actions in a paracrine or autocrine way. Our hypothesis was partially supported by another independent study, which demonstrated that the presence of resistin appeared in Kupffer cells, in a subset of endothelial cells, and occasionally in fibroblast-like cells [36]. Previously it was also suggested that hepatocyte could produce resistin [33], but the results from Szalowska’s group [36] and our group could not confirm this observation.

## Conclusions

In summary, the present study suggests that resistin is overexpressed in liver of patients with NASH, resistin correlates with the severity of liver necroinflammation and fibrosis. Hepatic resistin may be primarily produced by perisinusoidal cells (such as Kupffer cells and HSCs) in human NASH. As no specific receptors for resistin have been identified, the precise cellular or molecular mechanism of resistin contributing to NAFLD development and its disease progression needs further investigations.

## Abbreviations

NAFLD: Nonalcoholic fatty liver disease; NASH: Nonalcoholic steatohepatitis; TNF-ɑ: Tumor necrosis factor ɑ; FIZZ-3: Found in inflammatory zone-3; BMI: Body mass index; WHR: Waist-hip ratio; ALT: Alanine aminotransferase; AST: Aspartate aminotransferase; GGT: γ-Glutamyl transferase; TG: Triglyceride; TC: Total cholesterol; HDL-C: High-density lipoprotein cholesterol; LDL-C: Low-density lipoprotein cholesterol; HOMA-IR: Homeostasis model assessment of insulin resistance; NAS: NAFLD Activity Score; PCR: Polymerase chain reaction; GAPDH: Glyceraldehyde-3-phosphate dehydrogenase; SQ: Semi-quantitative; Hpf: High power field; CK-18: Cytokeratin-18; SD: Standard deviation; OR: Odds ratio; CI: Confidential index; HSC: Hepatic stellate cell.

## Competing interests

The authors have no competing of interests to declare.

## Authors’ contributions

CYZ designed the study and critically revised the manuscript. CS performed the major role of doing experiments, analyzed the data, and wrote the manuscript. WW, YDW, WC, LZ, and HS performed experiments and statistical analysis. ML, WYY and RJ managed the patients and colleted the data. JG collected the liver tissues. All authors have read and approved the final version of manuscript.

## Pre-publication history

The pre-publication history for this paper can be accessed here:

http://www.biomedcentral.com/1471-230X/14/39/prepub
